# Antiseptic effect of low-concentration povidone-iodine applied with a depot device in the conjunctiva before cataract surgery

**DOI:** 10.1038/s41433-018-0198-9

**Published:** 2018-08-31

**Authors:** Simon Wass, Grethe Albrektsen, Maria Tjåland Ødegård, Mari Sand, Dordi Austeng

**Affiliations:** 1 Department of Neuromedicine and Movement Science, Norwegian University of Science and Technology, Trondheim, Norway; 2 Department of Ophthalmology, St. Olavs Hospital, Trondheim University Hospital, Trondheim, Norway; 3 Department of Public Health and Nursing, Faculty of Medicine and Health Sciences, Norwegian University of Science and Technology, Trondheim, Norway

## Abstract

**Purpose:**

Examine the antiseptic effect of long-term low-concentration (0.3%) povidone-iodine (PI) before cataract surgery.

**Setting:**

St. Olavs Hospital, Trondheim University Hospital, Trondheim, Norway.

**Design:**

Single-armed prospective clinical study.

**Methods:**

Repeated measure of preoperative conjunctival samples from 51 participants were obtained the day before surgery (T1), the day of surgery after treatment with ophthalmic NSAID (T2), and after additional treatment with low-concentration PI (T3) given by placing a pledget soaked in a mix of eye-drops in fornix inferior for 20 min.

**Results:**

Before surgery, and before any type of treatment (T1), bacterial growth (≥5 BC) in the conjunctiva was identified in 36 (66.7%) of the participants. After treatment with ophthalmic NSAID (T2), and after additional treatment with low-concentration PI (T3), bacteria were identified in 31 (60.8%) and 12 (23.4%) participants, respectively. All except one of the participants with a measurable change from T2 to T3 (*n* = 31, 60.8% of total sample), experienced a decrease in number of bacterial colonies (BC) after treatment with low-concentration PI (96.8 vs. 3.2%, *p* < 0.001). A complete removal of bacteria was seen in 20 (*n* = 31, 64,5%) of the colonized participants after treatment with PI.

**Conclusions:**

Preoperative treatment with long-term, low-concentration PI applied via a depot device in fornix inferior, seem to be an easy and effective way to reduce the number of BC in the conjunctiva.

## Introduction

Endophthalmitis is a feared postoperative complication to cataract surgery with an incidence proportion found to be approximately 0.05% in two large studies from Sweden and Japan, respectively [1, 2]. In a study from USA, 0.14% of cataract surgery participants suffered from acute postoperative endophthalmitis [3]. Although rare, prevention of this bacterial induced complication is of great importance since it may lead to poor visual outcome even with appropriate treatment. Bacterial contamination to the eye originates from the eyelid, periorbital skin, and conjunctiva [4, 5], but the conjunctiva is assumed to be the primary source to the commensal bacteria that increases the risk of developing endophthalmitis [6]. Fluorescein-stained conjunctiva fluid has been observed to enter the anterior chamber during cataract surgery [7, 8, 9], supporting the theory that commensal bacteria is involved in the etiology of endophthalmitis. A reduction in preoperative conjunctival bacterial load are thus of outmost importance to reduce the risk of this severe complication.

Povidone-iodine (PI) is the most used antiseptic substance since it was introduced in the 1950s and has been applied within ocular surgery since the 1990s. Together with improved and sterile surgery technique [6] and per-operative intra-cameral injection of antibiotic [10, 11], preoperative disinfection with PI is of profound significance to avoid postoperative infections [12]. PI is highly effective against a wide spectrum of microbes, there is no observed resistance [13] and it does not seem to influence healing of wounds [14]. The tissue toxicity of PI depends on the concentration, and a higher concentration (>1%) seems to damage the corneal epithelial cells in rabbit eyes [15]. In humans, a concentration level at 10% PI has most often been used when disinfecting the eyelid and conjunctiva before ocular surgery. However, one clinical trial indicated that a 5% concentration PI is less toxic and equally effective as 10% concentrations in reducing bacteria, even with a shorter exposure time [16]. In vitro studies have shown bactericidal effect of even lower concentrations of PI [17. 18], although this effect has not been reproduced in vivo [19]. Results from another clinical study, however, suggested a substantial bactericidal effect of repeated irrigation of low-concentration PI (0.25%) during cataract surgery, reducing anterior chamber contamination [20]. For low-concentration PI to obtain an antiseptic effect in vivo, it appears that both a sufficient amount of the drug and sufficient time of exposure are essential to enable the mixture to infiltrate and disinfect the complex surface structure of the conjunctiva.

The aim of the present study was to investigate the antiseptic effect of long-term low-concentration (0.3%) PI on the number of bacterial colonies (BC) before cataract surgery. The standard preoperative procedure prior to cataract surgery at the hospital where the study was conducted (St. Olavs Hospital, Trondheim, Norway), consists of a pledget soaked in a mixture of eye drops including low-concentration PI, placed in fornix inferior for 20 min. In addition, we rinse the conjunctiva and the surrounding skin with PI 5% 2 min before surgery. The idea is that the pledget acts as a depot, prolonging the exposure time of low-concentration PI. The procedure intends to give a dilated pupil [21] and a reduction in bacterial load before cataract surgery. This concentration of PI, applied with this administration technique to our knowledge is not described before. We started using this preoperative procedure from 1st March 2007. Between 1st March 2007 and 28th February 2018 more than 18,000 cataract operations have been performed at St. Olavs hospital and we have had 1 postoperative endophthalmitis due to cataract surgery. This gives a postoperative endophthalmitis incidence of 0.0055% which is rather low, and we suspect the pledget to be one important reason for the low incidence ratio. However, whether and to what extent it reduces bacterial load in the conjunctiva, have not yet been explored. A secondary aim was to examine what type of bacteria that could be identified before and after treatment with low concentration PI.

## Method

### Study population

**Fig. 1:**
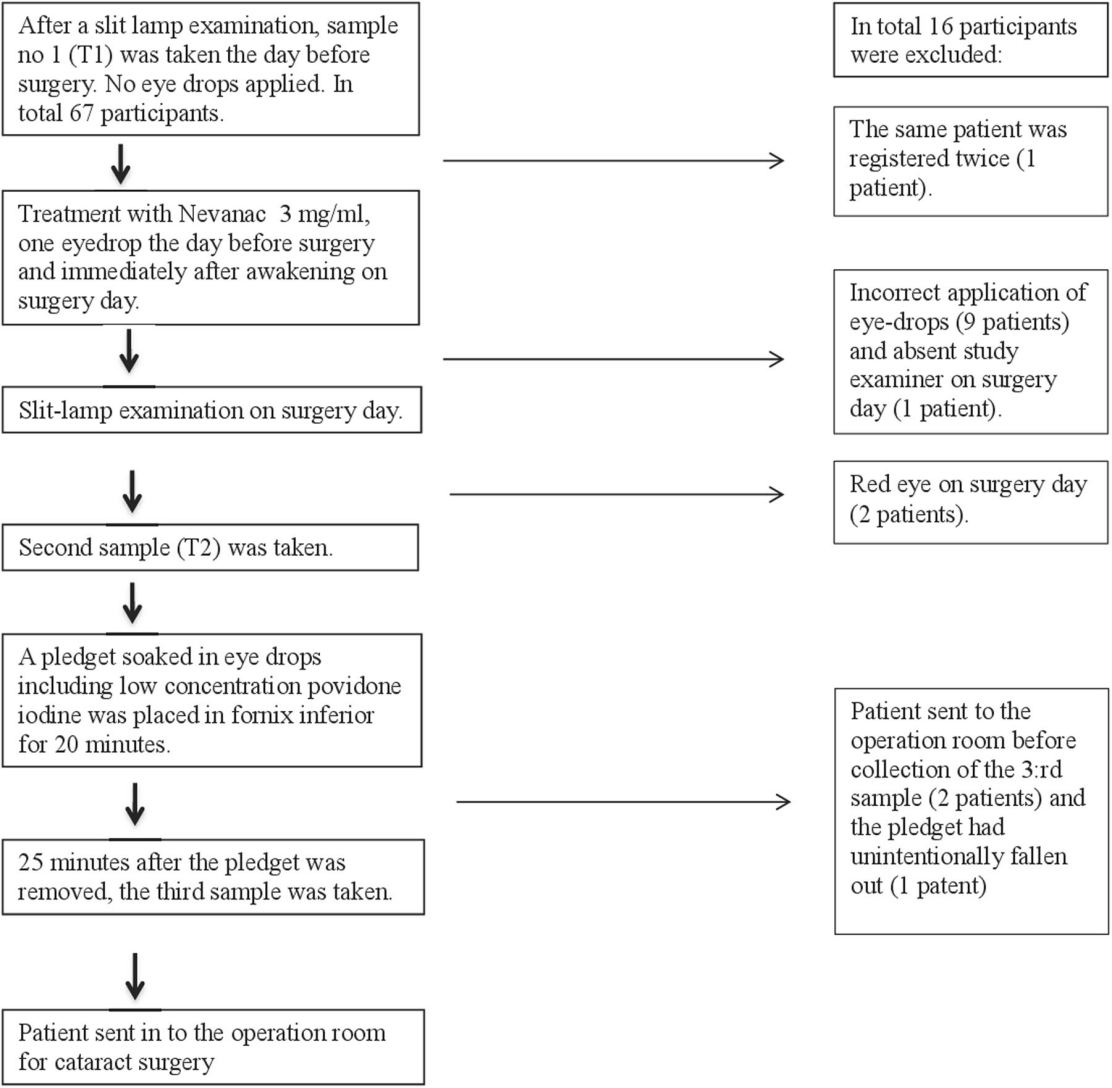
Flow-chart of inclusion and exclusion of participants to the study

Participants referred to cataract surgery at the Department of Ophthalmology, St. Olavs Hospital, Trondheim University Hospital, during November 2015 to September 2016, were invited to participate in the study. Information about the study was given both oral and written. The inclusion criteria were: adequate cognitive state to understand the given information. The exclusion criteria were: allergy for eye drops, use of any kind of eye drops/ointment, use of systemic antibiotics, or use of corticosteroids in the last 2 weeks, ongoing ocular or orbital infection or inflammation, and under 60 years of age. Informed consent for participation was ascertained from 67 participants, but 16 were later excluded due to deviations from the study protocol, leaving us with 51 participants eligible for analysis (Fig. 1), 30 women and 21 men. The mean age was 74.6 years, ranged from 61 to 88. The study was approved by The Norwegian Medicines Agency (EudraCT number: 2015-002292-20) and the Regional Ethical committee (2015/320/REK vest).

### Timing of sampling

The trial used repeated measure design where every single participant was exposed to every single treatment, including the control. To be able to evaluate the effect of treatment, a preoperative conjunctival sample from 51 participants was obtained from the fornix inferior of the eye at three different time points prior to cataract surgery (T1, T2, and T3).

T1–the first sample was taken the day before surgery. At this point of time no eye drops had been applied to the participant´s eye. This was the control sample.

T2–the second sample was taken on the day of surgery after standard treatment with ophthalmic NSAID (Nepafenac 3 mg/ml); one eye drop in the evening the day before and one eye drop the same morning.

T3–the third sample was taken after treatment with long-term low-concentration PI. Standard procedures were followed, with application of a pledget soaked in a mix of eye drops including PI (Table 1), placed in fornix inferior for 20 min. The concentration of PI in the mixture was 0.3% (3.39 mg/ml), estimated from the size of the eye drops in the mixture, estimation performed by the Hospital pharmacy St. Olavs Hospital, Trondheim University Hospital. The third sample was taken 25 min after removal of the pledget with low-concentration PI, immediately thereafter the participant was sent to the operating room.

**Table 1:** Content of the preoperative eye-drop mixture sufficient for 6 pledgets

Active substance	Concentration	Number of drops	Producer
Cyclopentolat/hydrochloride (minims)	1%	2	Bausch & Lomb, UK
Phenylephrine/hydrochloride (minims)	10%	3	Bausch & Lomb, UK
Oxybuprocaine/hydrochloride (minims)	0.4%	10	Bausch & Lomb, UK
Diclofenac^a^ (minims)	0.1%	4	Laboratoires THEA, France
Povidone iodine (minims)	5%	2	Bausch & Lomb, UK
Total number of eyedrops		**21**	

^a^Brand name Voltaren Optha

### Use of a pledget as depot device

The pledget, was “Spongostan dental–Absorbable Haemostatic Gelatin Sponge” (Ethicon, Inc. Somerville, USA), chosen because of its water-insoluble and malleable characteristics. Spongostan comes in size 10 × 10 × 10 mm, that manually and under sterile conditions was cut in half and put in the mixture, all of it kept in a sterile container. The mixture was sufficient for six pledgets. When the pledgets had absorbed all the mixture in the container, one pledget was placed in fornix inferior for 20 min. To get maximal effect of the mydriatic drops, the participants then had to wait another 25 min before surgery.

### Sampling procedure

Before taking the first sample (T1), the external part of the eye and anterior segment were examined in the slit lamp, to make sure there was no ongoing infection or inflammation. The examination was repeated before the second sample (T2). Two participants felt discomfort and had conjunctival injection the day after the first sample was taken (T1). Their cataract operation was postponed, and they were excluded from the study (Fig. 1).

The examiner then took the samples from fornix inferior with an e-Swab (Collection and preservation of aerobic, anaerobic and fastidious bacteria. Copan, Brescia Italy). While the participant was asked to look upward, the conjunctiva of the inferior fornix was exposed by a gentle pull in the skin just below the lower eyelid. The dry brush of the e-Swab was then placed in the inferior fornix and gently twirled 3 times, trying not to get in contact with the eyelids and the surrounding skin to avoid contamination. The sample was immediately placed in the e-Swab-medium for bacterial preservation and put in a refrigerator where it was kept for 1–6 h until it was manually transported to the laboratory. Before the third sample (T3) was collected, the participants were asked about perceived discomfort and looked for signs of ocular irritation. If none of these adverse reaction was observed, for the further treatment, the third sample was taken.

### Laboratory procedure

At the laboratory, under strict sterile conditions, each sample was placed on a mini-shaker (IKA MS 3 basic. Sigma-Aldrich, USA) for 10 s with 2500 rounds per minute, in order to loosen the bacteria from the e-Swab brush to the e-Swab medium. With a pipette, 100 microliters were manually plated on one blood agar plate and 100 microliters on one chocolate agar plate, both non-selective and non-enriched (both agar plates were produced by the Department for medical microbiology, St. Olavs Hospital, Trondheim, Norway). The blood agar and chocolate agar was then placed in an incubator (Forma Steri-cycle CO2 Incubator, model no. 371, Thermo Scientific Inc.) for 2 days before they were analyzed by two co-workers at the laboratory, counting the number of bacteria per milliliter and typing each bacterium. Since we wanted to quantify the BC in numbers per milliliter, and the total plated volume was 200 microliters (100 microliter on each plate), we multiplied the obtained number of BC on the plates by five to get the intended unit. The examination in the slit-lamp, the collection of samples, and the work at the laboratory plating the sample to the agar-plates was made by the same person; a physician at the Department of Ophthalmology, St. Olavs Hospital, Trondheim.

### Statistical analysis

The non-parametric Friedmans test was applied to test for difference in the number of BC between the three different sampling time points (T1, T2, T3). This is a test for overall difference in distributions of ranks, after having ordered the repeated measurements (rank 1–3) on individual level. Wilcoxon sign-rank test and the sign test, was applied to test for systematic change in a particular direction (increase or decrease) after treatment with ophthalmic NSAID (from T1 to T2), and after long-term low-concentration PI treatment (from T2 to T3). A chi-square test was applied to compare the proportion of participants with and without a change in number of BC, regardless of whether it was an increase or decrease in the number of BC. Moreover, the McNemar’s test was applied to test for change in presence/absence-status of bacteria between adjacent time points (original BC variable dichotomized, <5, i.e., 0 and ≥5, respectively).

## Results

**Fig. 2:**
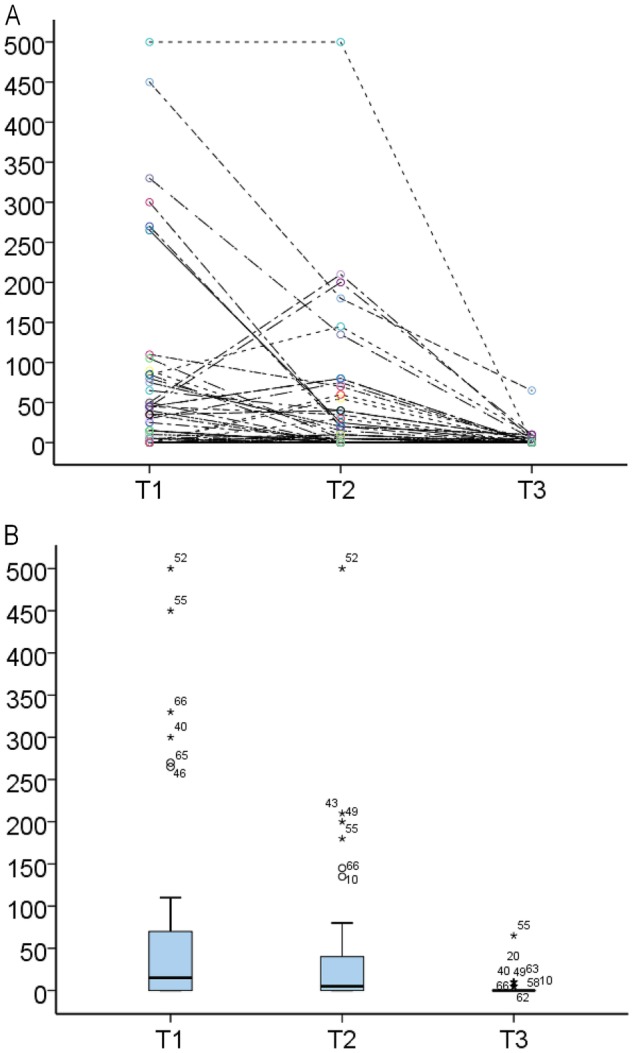
Number of bacterial colonies (BC) before treatment (T1), after treatment with ophthalmic NSAID (T2), and after additional treatment with low-concentration povidone-iodine (T3). **a** Individual time pattern. **b** Box-plot with median, quartiles and range in number of BC

The individual variation in number of BC was large, in particular before treatment (Fig. 2A) but both the number and spread in BC decreased, especially after treatment with low-concentration PI (Fig. 2B, Table 2). The median number of BC before treatment (T1), after treatment with ophthalmic NSAID (T2), and after treatment with long-term low-concentration PI (T3) was 15 (interquartile range 0–75), 5 (interquartile range 0–40), and 0 (interquartile range 0–65), respectively. The test for overall difference in the levels of bacterial load between the three sampling time points was highly significant (*p* < 0.001). The proportion of participants presenting with bacteria (no. of BC ≥5, any type) at each of these occasions was 66.7, 60.8, and 23.6%, respectively (Table 2). Nine different types of microorganisms were identified, all being part of normal bacteria flora (Supplemental Table 1). In 12 participants (23.5%), two or more bacterial species were found.

**Table 2:** Number (%) of bacterial colonies in the conjunctiva before treatment (T1), after treatment with NSAIDS (T2), and after additional treatment with low-concentration povidone-iodine (T3)

	Pre-treatment	Post-treatment	
	(T1)	NSAIDS (T2)	LC-PI (T3)
Number of BC			
Median (IQR^a^)	15 (0–75)	5 (0–40)	0 (0–0)
Min.-max.	0–500	0–500	0–65
Mean rank	2.40	2.20	1.40
*p*-value^b^			<0.001
BC category (*n*, %)			
0 (<5)	17 (33.3)	20 (39.2)	39 76.5)
5–45	18 (35.3)	19 (37.3)	11 21.6)
≥50	16 (31.3)	12 (23.5)	1 (2.0)

*BC* bacterial colonies, *IQR* interquartiles range, *LC-PI* low-concentration povidone-iodine

^a^Interquartile range given by 25 and 75% percentiles

^b^Friedman’s test for overall difference in distribution of no. of BC

Results from tests for systematic change in BC after treatment with NSAIDS and low concentration PI, respectively, are shown in Table 3. The proportion of participants that experienced a change in no. of BC (≥5, any direction, any level) after treatment with ophthalmic NSAID was considerably higher than the proportion of participants that did not experience a change (76.5% vs. 23.5%, *p* < 0.001). However, no significant systematic change was seen (*p* = 0.20), since both a decrease (61%) and an increase (38.5%) in no. of BC was seen among participants experiencing a change. The difference was more pronounced, though still not statistically significant (*p* = 0.068) when considering the magnitude of change, in addition to direction. In contrast, additional treatment with low-concentration PI led to a reduction in bacterial load in all except one of the participants that experienced a change (96.8% vs. 3.2%, *p* < 0.001), although the difference in the proportion of participants with and without a change did not differ significantly (60.8% vs. 39.1%, *p* = 0.12).

**Table 3:** Change in no. of bacterial colonies after treatment with NSAIDS (T2 vs. T1) and after additional treatment with low-concentration povidone-iodine (T3 vs. T2)

	NSAIDS	LC-PI
	T2 vs. T1	T3 vs. T2
Change in no. of BC		
Median (IQR^1^)	0 (−40, 5)	−5 (−35, 0)
Min.-max.	(−280, 160)	(−500, 5)
Mean rank		
Decrease (neg. rank)	21.7	16.4
Increase (pos. rank)	17.3	3.5
* p*-value^a^	0.068	<0.001
Change of ≥5 BC, any level (*n*, %)		
No change (any level)	12 (23.5)	20 (39.2)
Change, either direction (±5 BC)	39 (76.5)	31 (60.8)
* p*-value^b^	<0.001	0.12
Decrease (≤−5, neg. rank)	24 (61.5)	30 96.8)
Increase (≥+5, pos. rank)	15 (38.5)	1 (3.2)
* p*-value (≤−5. vs. ≥+5)^c^	0.20	<0.001
Change in BC-category (*n*, %)^d^		
No change (≥5 or 0)	34 (66.7)	30 (58.8)
Change (≥5 to 0, 0 to ≥5)	17 (33.3)	21 (41.2)
* p*-value^b^	0.024	0.26
No change, by BC-category		
BC not identified (0 to 0)	10 (29.4)	19 63.3)
BC identified (≥5 to ≥5)	24 (70.6)	11 (36.7)
Change, by direction of change		
Decrease (≥5 to 0)	10 (58.8)	20 (95.2)
Increase (0 to ≥5)	7 (41.2)	1 (4.8)
* p*-value^e^	0.63	<0.001

*BC* bacterial colonies, *LC-PI* low-concentration povidone-iodine

^a^Wilcoxon sign-rank test for change in specific direction (decrease vs. increase)

^b^Chi-square test for difference in 2 independent proportions (change vs. no change)

^c^Wilcoxon sign test for change in specific direction (decrease vs. increase)

^d^Dichotomized into presence (BC ≥ 5) or absence (BC = 0) of bacteria

^e^McNemar’s test for change in specific direction

A similar pattern was found when contrasting presence/absence of bacteria (Table 3). Whereas both a decrease (from presence to absence) and an increase (from absence to presence) in bacterial load were seen after treatment with ophthalmic NSAID (58.8% vs. 41.2%, *p* = 0.63), long-term low-concentration PI almost exclusively led to a complete removal of bacteria among participants who did experience a change (95.2% vs. 4.8%, *p* < 0.001). However, 11 participants (21.6% of total sample) remained with bacteria also after treatment with PI. The 20 participants with complete removal of bacteria amounted 64.5% (*n* = 31) of colonized participants. One participant without bacteria after ophthalmic NSAID presented with bacteria after treatment with low concentration PI. No adverse events were identified and no discomfort after low-concentration PI treatment was reported in any of the 51 participants.

## Discussion

In this study, pre-operative treatment with low-concentration PI reduced the number of BC in almost all participants that experienced a change in bacterial load. A rather large proportion of participants did not experience a change, but this also related to bacterial status before treatment, with approximately 40% being without bacteria. Application of a pledget as a depot for low-concentration PI in fornix inferior, appears to be an uncomplicated, inexpensive, and safe procedure for reducing the bacterial load in the conjunctiva before cataract surgery. The identified bacteria were all part of the normal bacterial flora.

Our results are consistent with those reported by Shimada et al. [20] who found an extremely low bacterial contamination rate in the anterior chamber at completion of surgery. In contrast to the pre-operative procedures applied in our study, however, they repeatedly irrigated the operative field with low-concentration PI (0.25%). Amount of low-concentration PI and time of exposure in the conjunctiva seem to be of importance for the diluted PI to infiltrate the complex surface structure of the conjunctiva. Prolonged exposure time can be ensured both through repeatedly irrigating the conjunctiva either pre-operative or per-operative, or by constant release of PI from a depot prior to surgery.

Results from a previous study suggests that free iodine is released more readily from its molecular complex in diluted PI solutions compared to concentrated solutions [17]. Free iodine is cytotoxic to the prokaryotic bacteria cell [22], and the higher the amount of free iodine, the higher the bactericidal effect. The maximal free iodine is previously found to be at a PI concentration of 0.7%, [13] and 0.1–1% PI is found to kill bacteria in vitro significantly faster compared with 2.5–10% PI. [17] One previous clinical study was, however, not able to reproduce this highly antiseptic effect of diluted PI in vivo when comparing 5 PI vs. 1% PI [19]. As mentioned by Ferguson et al., the discrepancies between the in vitro and in vivo results might be explained by the mode and the duration of application of PI [19]. Since the free iodine is released from the molecular complex until the complex is exhausted for iodine, the high bactericidal effect might be dependent of availability of a sufficient amount of low-concentration PI.

In the present study, no adverse events or discomfort were reported during or after treatment with low-concentration PI. This supports the statement from Trost, as well as Shimada et al., that low-concentration PI is safe for the ocular tissue [20, 23]. Consistent to previous findings, the present study indicates a strong antiseptic effect of low-concentration PI (0.3%), and that the short-lasting effect of low-concentration PI might be compensated by the pledget, serving as reservoir providing a continuous flow of low-concentration PI in the conjunctiva.

Standard preoperative treatment prior cataract surgery at St. Olavs hospital is treatment with Nepafenac 3 mg/ml since previous studies indicate it maintains mydriasis during surgery and prevent postoperative ocular pain, inflammation, and cystoid macular edema [24, 25, 26, 27]. Peroperative intracameral mydriatic injections have previously been shown to be as effective as mydriatic eyedrops, however mydriatic eyedrops are by many still considered to be the standard method for pupil dilation in cataract surgery [28]. In this study a two-sided beneficial effect using the pledget was demonstrated; the eye had a good and stable mydriasis, while reducing the bacterial load in the conjunctiva. Our experience is that this method gives a stable mydriasis present when the patient enters the operating room, thus saving operating time, as well as reducing bacterial load and likely postoperative endophthalmitis.

Our finding of bacteria in 67% of the participants before treatment is consistent with findings in a previous study [6]. Although we did not manage to identify any bacteria in 33% of the participants, this does not necessary imply sterility in those eyes, an argument transferable to all three samples collected. One participant had an increase in bacterial load after treatment with povidone-iodine 0.3%. In this case, zero colonies were identified in sample one and two, and in sample three, five colonies Coagulase-negative Staphylococci were identified after treatment with the pledget. Considering the type of bacteria and the moderate number of colonies, we suspect contamination from the surrounding skin during sample collection to be the reason for the increase in bacteria from T2 to T3. The patient did not have a red eye and was operated without complications. The choice of culturing method is always an issue when quantifying amount and type of bacteria. We applied non-selective non-enriched media with an incubation time of 48 h when culturing the samples. A longer time in the incubator might reveal additional slow growing bacteria, but at the same time increase the risk of false positive results. Regardless of the media used, there is always a potential for false positive results present [29]. An increase in BC was seen in 15 participants between T1 and T2, and two participants were excluded after slit-lamp examination on surgery day because of a red eye. This may indicate a possible increase in conjunctival bacterial load after taking the samples, since a mechanical manipulation of the conjunctiva and eyelids during sample collection might promote bacterial release. Although not applied in our study, using control cultures or a control group would have been valuable when trying to determine the contamination rate. The same examiner was collecting all the samples in this study, making the collecting procedure consistent for all samples taken.

A limitation of the study is rather the low sample size. The results are, however, promising in reducing the bacterial load in the conjunctiva, and seem to bolster the idea of a highly bactericidal effect of low-concentration PI also in vivo, given sufficient availability to low-concentration PI in the conjunctiva. Costs and efficacy of shorter and longer exposure time, combined with higher or lower concentration of PI, as well as evaluation of different procedures for administration of treatment, needs to be explored in future studies.

### Summary

#### What was known before

- Free iodine acts cytotoxic to the prokaryotic bacteria cell.
- Low-concentration of povidone-iodine in vitro is more bactericidal than higher concentrations of povidone-iodine because free iodine is more readily released from its molecular complex in diluted solutions.
- After cataract operation, lower contamination rate is found in anterior chamber when repeatedly irrigating with low-concentration Povidone-Iodine.

#### What this study adds

- A pledget with low-concentration povidone-iodine placed in fornix inferior for 20 min, seem to work as a depot reducing the conjunctival bacterial load.
- This way of preoperative treatment was easy to use and gave no local irritation.

## Electronic supplementary material

Supplemental Table1
