# Supplementary material for: Antiseptic effect of low-concentration povidone-iodine applied with a depot device in the conjunctiva before cataract surgery
Source: Eye (Lond). 2018 Aug 31;32(12):1900–7. doi: 10.1038/s41433-018-0198-9 (PMC6292903; doi:10.1038/s41433-018-0198-9)
Supplement: Supplementary file 1 — Supplemental Table1 [file 41433_2018_198_MOESM1_ESM.docx]

**Supplemental Table 1.**

Micro-organisms identified from participants

| **Micro-organisms** | **Number of participants with microorganisms present** | | |
| --- | --- | --- | --- |
|  | Before treatment | After Nepafenac- treatment | After PI-treatment |
| Coagulase-Negative Staphylococci | 32 | 29 | 11 |
| Alpha-haemolytic streptococcus | 4 | 3 | 1 |
| Staphylococcus Aureus | 3 | 2 | 0 |
| Corynebacterium Pseudodiphteriticum | 1 | 1 | 0 |
| Moraxella Osloensis | 0 | 1 | 0 |
| Propionebacterium acnes | 1 | 0 | 0 |
| Actinomyces Oris | 1 | 1 | 0 |
| Actinomyces Neuii | 1 | 0 | 0 |
| Corynebacterium Striatum | 1 | 0 | 0 |
|  |  |  |  |
